# Quantification of perflutren microsphere contrast destruction during transit through an ex vivo extracorporeal membrane oxygenation circuit

**DOI:** 10.1186/s40635-016-0079-0

**Published:** 2016-03-11

**Authors:** David G. Platts, Charles McDonald, Kiran Shekar, Darryl J. Burstow, Daniel Mullany, Marc Ziegenfuss, Sara Diab, John F. Fraser

**Affiliations:** Department of Echocardiography, The Prince Charles Hospital, Rode Rd., Chermside, Brisbane, Queensland 4032 Australia; Critical Care Research Group, The Prince Charles Hospital, Rode Rd., Chermside, Brisbane, Queensland 4032 Australia; The University of Queensland, Brisbane, Queensland Australia; Adult Intensive Care Service, The Prince Charles Hospital, Rode Rd., Chermside, Brisbane, Queensland 4032 Australia; Queensland Advanced Heart Failure and Cardiac Transplant Unit, Department of Echocardiography, The Prince Charles Hospital, Rode Rd., Chermside, Brisbane, Queensland 4032 Australia

**Keywords:** Extracorporeal membrane oxygenation, Contrast echocardiography, Oxygenator

## Abstract

**Background:**

Echocardiography is a key investigation in the management of patients on extracorporeal membrane oxygenation (ECMO). However, echocardiographic images are often non-diagnostic in this patient population. Contrast-enhanced echocardiography may overcome many of these limitations but contrast microspheres are hydrodynamically labile structures prone to destruction from shear forces and turbulent flow, which may exist within an ECMO circuit. This study sought to evaluate microsphere destruction (utilising signal intensity as a marker of contrast concentration) during transit through an ECMO circuit.

**Methods:**

Activated Definity® contrast was diluted to 50 ml with normal saline and infused into a crystalloid primed ex vivo ECMO with a Quadrox oxygenator at 150 ml/h. Imaging was performed on pre- and post-pump head/oxygenator sections of the circuit using a Philips iE33 scanner and S5-1 transducer. Five-millimetre regions of interest were placed in the centre of the ultrasound field. Average signal intensity (decibels) was calculated at speeds of 1000, 2000, 3000 and 4000 rpm and then repeated with an infusion rate of 300 ml/h. The oxygenator was then spliced out of the circuit and the measures repeated.

**Results:**

There was a significant reduction in contrast concentration during passage through the ECMO circuit at all speeds (with higher pump head speeds resulting in greater microsphere destruction). In a circuit with an oxygenator, relative decrease in signal intensity was 21.4 versus 5.2 % without an oxygenator. There was significant destruction of contrast microspheres during passage through the ECMO circuit at all pump head speeds. An oxygenator contributed to microsphere destruction at a significantly greater level than the pump head alone. There was no significant difference in mean signal intensity reduction in the circuit between an infusion of 150 or 300 ml/h (3.5 ± 3.2 versus 3.6 ± 2.5 dB, respectively, *p* = 0.79).

**Conclusions:**

Flow of contrast through an ECMO circuit results in significant destruction of microspheres. Circuits with an oxygenator result in significantly greater levels of contrast destruction than by the pump head alone. Clinicians should be cognisant of the relationship between ECMO circuit configurations, pump head speed and contrast destruction when performing a contrast-enhanced echocardiogram in patients supported with ECMO.

**Electronic supplementary material:**

The online version of this article (doi:10.1186/s40635-016-0079-0) contains supplementary material, which is available to authorized users.

## Background

Extracorporeal membrane oxygenation (ECMO) is a modified form of cardiopulmonary bypass used to provide cardiac and/or respiratory support in patients that have not responded to maximal medical support [1–5]. The use of ECMO is increasing [6], and echocardiography plays a fundamental role throughout the care of a patient supported on ECMO [7, 8]. However, provision of ECMO usually takes place within a critical care complex and assessment of these patients using transthoracic echocardiography can be challenging due to sub-optimal image quality in up to 25 % of patients. Reasons for this include the inability to position the patient to optimise acoustic windows, sub-optimal lighting conditions, invasive ventilation and the presence of surgical drains or sites limiting scanning planes. The administration of a contrast agent during echocardiography can help minimise these non-diagnostic echocardiograms [9–11].

However, these contrast microspheres are hydrodynamically labile structures and are prone to destruction from shear forces and turbulent flow, which exist within an ECMO circuit. This destruction within an ECMO circuit has never been confirmed nor quantified. Reduced contrast concentration due to microbubble destruction would alter ultrasound backscatter signal intensity and hence may affect ultrasound image quality and reduce accuracy of the test. The aim of this study was to quantify the degree of microsphere destruction, utilising signal intensity as a marker of contrast concentration, during transit through an ex vivo ECMO circuit using quantitative echocardiography. A secondary aim was to assess the impact of an oxygenator on bubble destruction within an ECMO circuit.

## Methods

An ex vivo ECMO circuit was primed and operated at multiple pump head speeds. An echocardiographic contrast agent was administered via an infusion, and echocardiographic images were obtained pre- and post-pump head to assess signal intensity, used as a surrogate marker for contrast concentration.

### ECMO circuit

**Fig. 1:**
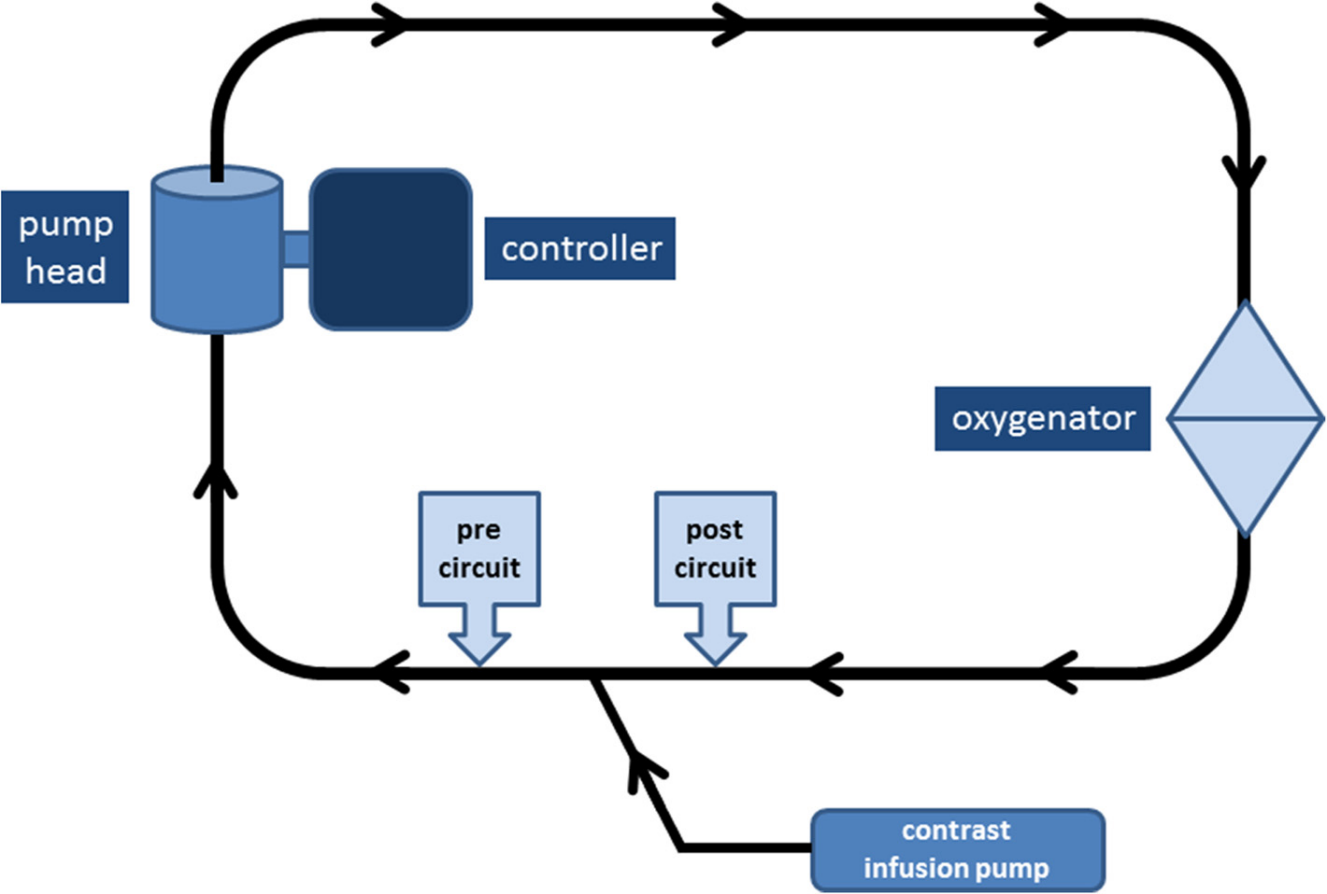
Schematic diagram of the ECMO circuit, with *arrows* indicating direction of flow

The ECMO circuit consisted of a bioline-coated Quadrox PLS oxygenator plus RF32 Rotaflow centrifugal pump head connected to a Rotaflow drive console (Maquet Cardiopulmonary AG, Hirrlingen, Germany) with no reservoir. The circuit was primed with 0.9 % sodium chloride (Baxter Healthcare Pty Ltd, Toongabbie, NSW, Australia), 30 ml of 8.4 % sodium bicarbonate (Pfizer Australia, West Ryde, NSW, Australia) and warmed to 37 °C. The oxygenator was filled with room air (FiO_2_ of 21 %). Sideport tubing provided access for administration of the contrast agent prior to the pump head. The ECMO circuit was run at four pump head speeds: 1000, 2000, 3000 and 4000 rpm, during an infusion of an echocardiographic contrast agent. Figure 1 is a schematic of the circuit design (arrows indicate direction of flow).

### Contrast agent

Definity® (Lantheus Medical Imaging, Billerica, MA, USA) contrast agent is a perflutren microsphere with a mean diameter of 1.1–3.3 μm. It consists of a central core of octofluoropropane gas, a high molecular weight (188), biologically inert gas and a tri-lipid outer shell [12]. The inactivated ampoule of Definity contrast consists of the clear liquid lipid shell below and the gaseous core above. It requires activation in a VialMix® device, which agitates the ampoule at 4530 ± 100 oscillations per minute for 45 s. This generates a solution of perflutren microsphere bubbles, which have an opaque, milky white appearance. Activation results in 1.3 ml of contrast. The activated contrast was then diluted to 50 ml with normal saline and infused using an Alaris® GH Plus (CareFusion, San Diego, CA, USA) infusion pump. Contrast was infused at two different rates, 150 and 300 ml/h. These rates were selected because 150 ml/h is the typical starting rate for a 50-ml dilution and 300 ml/h is a typical infusion rate for technically difficult patients in the critical care complex.

### Image acquisition and analysis

A 5-millimetre (mm)-thick silicon ultrasound stand-off was placed between the ultrasound transducer and ECMO circuit tubing. A S5-1 ultrasound transducer and Philips iE33 ultrasound scanner were used for image acquisition. Contrast-specific imaging was activated, utilising low mechanical index and power modulation imaging. The scanning settings were constant for all acquired images: transducer frequency of 2 MHz, gain of 40 %, compression at 50, depth of field of 5 cm, frame rate of 39 Hz and a mechanical index of 0.1. Following an infusion of contrast for 2 min, 2-s ultrasound clips were then acquired at two separate sites within the ECMO circuit. As such, each data point consisted of 78 ultrasound frames for analysis. Two-second ultrasound clips were then acquired at two sites within the ECMO circuit following an infusion of Definity contrast. The first site (labelled ‘pre-circuit’) was located 10 cm after the site of entry of contrast into the circuit but before the pump head. The second site (labelled ‘post-circuit’) was located at the end of the circuit and 10 cm before the site of entry of contrast into the circuit.

Contrast images were acquired at the four different pump head speeds (1000, 2000, 3000 and 4000 rpm), during an infusion of contrast at 150 ml/h. This was then repeated at a contrast infusion rate of 300 ml/h. Following acquisition of these images, the oxygenator was removed from the circuit and contrast was re-infused into the circuit. A repeat set of images were acquired at the four pump head speeds (1000–4000) and at the two different infusion rates (150 and 300 ml/h).

**Fig. 2:**
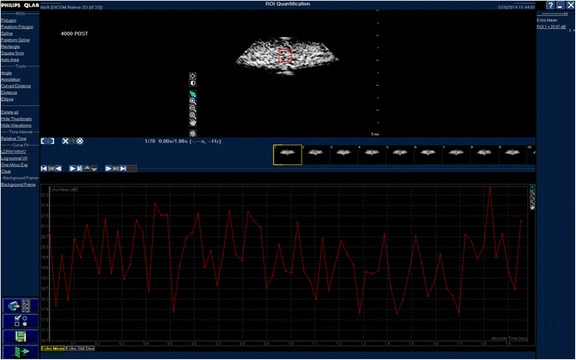
Example of a contrast signal intensity graph (measured in decibels) over 2 s with a central 5 mm^2^ region of interest

The images were transferred to a separate workstation and analysed using QLAB quantification software (Philips Medical Systems, Amsterdam, Netherlands). A pre-set 5-mm square region of interest (ROI) was placed in the centre of the acquired ultrasound image. The mean signal intensity (measured in decibels) was calculated over this 2-s clip. The mean signal intensity over this time interval was used as a surrogate of microbubble contrast concentration. Figure 2 is an example of a contrast signal intensity graph (measured in decibels) over 2 s with a central 5 mm^2^ region of interest.

### Statistical analysis

Contrast signal intensity for each view, pre and post, the ECMO circuit was expressed as mean ± one standard deviation. Comparison between the continuous variables was performed using the paired Student’s *t* test for pre- versus post-circuit analysis and unpaired Student’s *t* test for full circuit versus no oxygenator comparison. A *p* value of <0.05 was considered as statistically significant. Statistical analysis was performed using MedCalc version 10.0 (Mariakerke, Belgium).

## Results

Echocardiographic imaging was technically feasible at both locations (pre-circuit and post-circuit) at all ECMO speeds. There was a significant reduction in contrast concentration during passage through the ECMO circuit at all speeds. The mean signal intensities ± 1 standard deviation (measured in decibels) pre- and post-circuit for the combined speed data (full circuit and no oxygenator in the circuit) are displayed in Table 1. There was a significant reduction in signal intensity (mean of 5.4 dB) for combined data in a full circuit (from 25.3 ± 1.9 to 19.9 ± 2.6, *p* < 0.0001). There was a smaller but still significant reduction in signal intensity (mean of 1.6 dB) for combined data in a circuit with no oxygenator (from 30.8 ± 2.4 to 29.2 ± 2.0, *p* < 0.0001). In a circuit with an oxygenator, the relative decrease in signal intensity was 21.4 %, whilst in a circuit with no oxygenator, the relative decrease in signal intensity was 5.2 %. There was no significant difference in mean signal intensity variation in the circuit between an infusion of 150 or 300 ml/h (3.5 ± 3.2 versus 3.6 ± 2.5 dB, respectively, *p* = 0.79).

**Table 1:** Mean signal intensities (measured in decibels) pre- and post-circuit for the combined speed data (with and without an oxygenator in the circuit)

	Pre-circuit (dB)	Post-circuit (dB)	Difference	*p* value
Oxygenator 150 ml/h	24.6 ± 1.7	18.9 ± 2.4	5.7 ± 3.0	*p* < 0.0001
Oxygenator 300 ml/h	25.9 ± 1.9	20.8 ± 2.6	5.1 ± 2.1	*p* < 0.0001
Oxygenator combined	25.3 ± 1.9	19.9 ± 2.6	5.4 ± 2.6	*p* < 0.0001
No oxygenator 150 ml/h	30.9 ± 1.3	29.6 ± 1.4	1.3 ± 1.5	*p* < 0.0001
No oxygenator 300 ml/h	30.6 ± 3.1	28.8 ± 2.5	1.8 ± 1.8	*p* < 0.0001
No oxygenator combined	30.8 ± 2.4	29.2 ± 2.0	1.6 ± 1.7	*p* < 0.0001

**Fig. 3:**
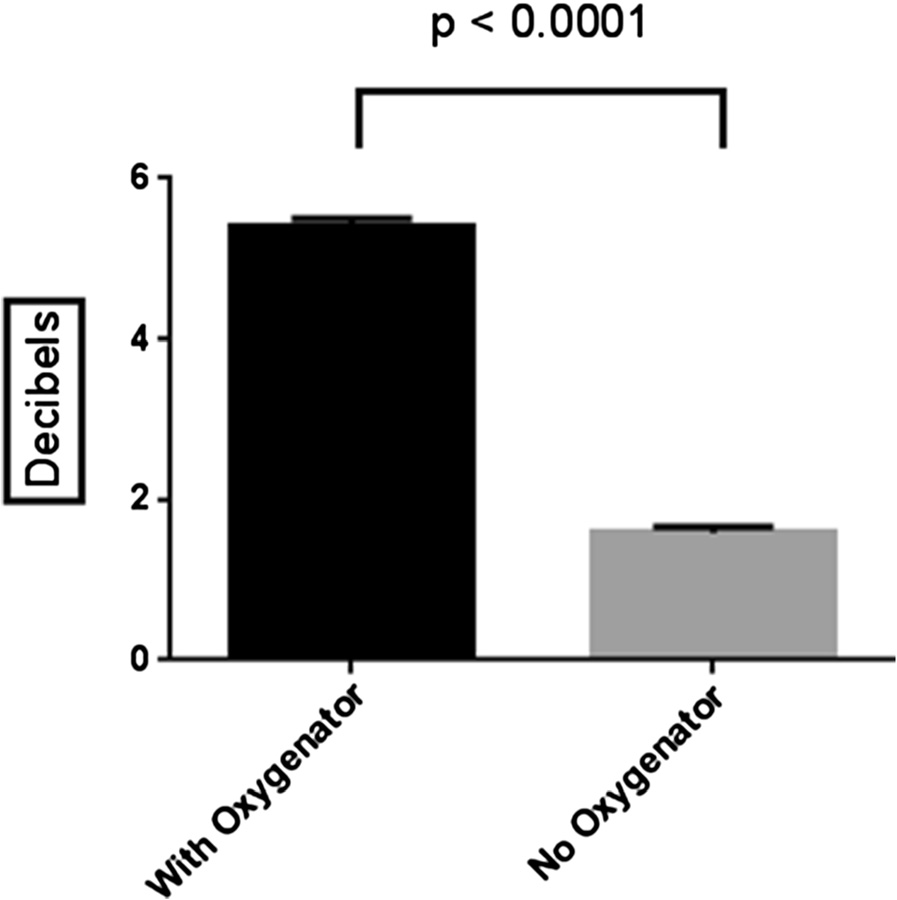
The mean difference in signal intensity (decibels) between a circuit with and without an oxygenator (combined data)

**Fig. 4:**
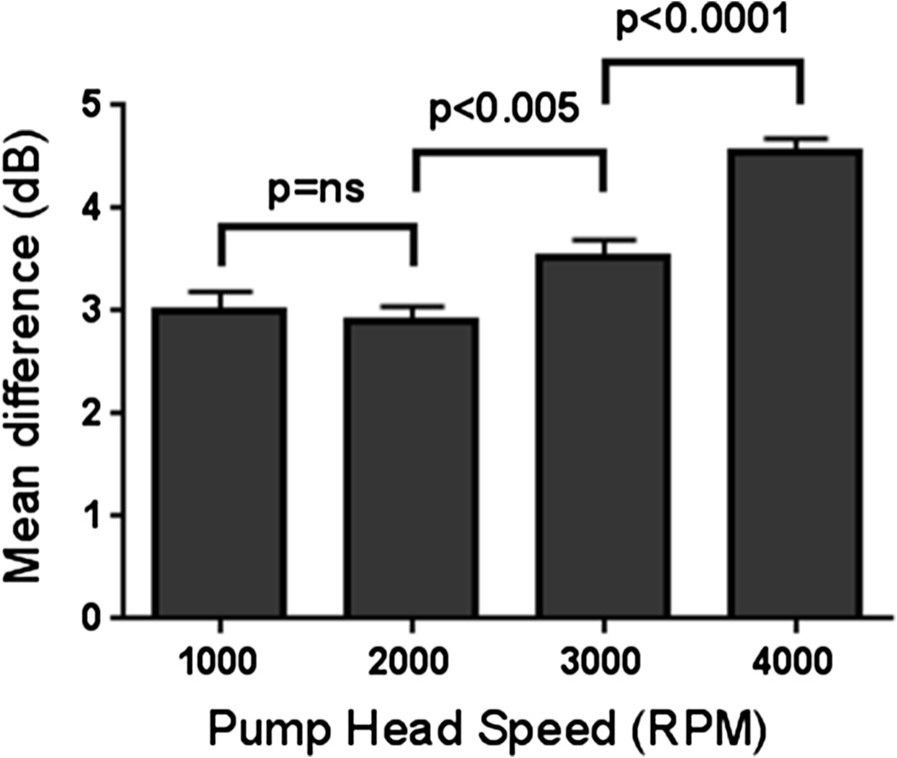
Mean difference (combined data) in signal intensity across the ECMO circuit at different pump head speeds (1000–4000 rpm). *ns* not significant

**Fig. 5:**
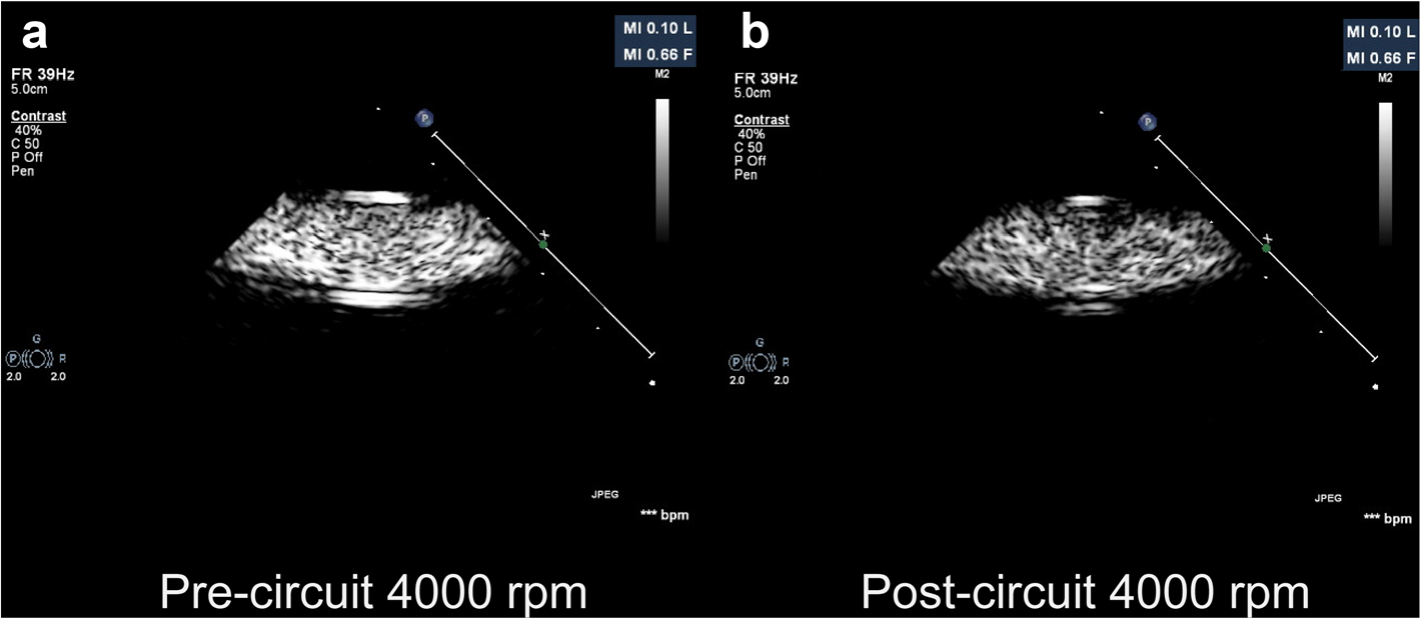
**a**, **b** Pre-ECMO and post-ECMO contrast echocardiographic images. Note the reduced signal intensity in the post-ECMO image

The mean difference in signal intensity between a circuit with and without an oxygenator is displayed in Fig. 3. The mean difference in signal intensity at the four pump head speeds and two infusion rates in circuits with and without an oxygenator is displayed in Table 2. At all pump head speeds, there was significantly greater microbubble destruction in an ECMO circuit with an oxygenator, compared to a circuit without an oxygenator. Mean difference in signal intensity for combined data (circuits with and without an oxygenator and at both infusion rates) at all four pump head speeds is displayed in Fig. 4. Figure 5a, b demonstrates examples of pre-circuit and post-circuit contrast echocardiographic images, respectively. Additional file 1: Video 1 and Additional file 2: Video 2 are the corresponding 2-s acquired clips.

**Table 2:** Mean difference in signal intensity (measured in decibels) at the four pump head speeds and two infusion rates in circuits with and without an oxygenator

	Mean difference (dB)		Mean difference (dB)	*p* value
1000 rpm oxygenator 150 ml/h	4.2 ± 2.6	1000 rpm no oxygenator 150 ml/h	1.4 ± 1.3	*p* < 0.0001
2000 rpm oxygenator 150 ml/h	4.5 ± 2.1	2000 rpm no oxygenator 150 ml/h	0.6 ± 1.6	*p* < 0.0001
3000 rpm oxygenator 150 ml/h	7.1 ± 2.4	3000 rpm no oxygenator 150 ml/h	0.7 ± 1.0	*p* < 0.0001
4000 rpm oxygenator 150 ml/h	6.7 ± 2.5	4000 rpm no oxygenator 150 ml/h	2.5 ± 1.0	*p* < 0.0001
1000 rpm oxygenator 300 ml/h	5.9 ± 2.7	1000 rpm no oxygenator 300 ml/h	0.6 ± 2.3	*p* < 0.0001
2000 rpm oxygenator 300 ml/h	3.9 ± 2.0	2000 rpm no oxygenator 300 ml/h	2.5 ± 1.6	*p* < 0.0001
3000 rpm oxygenator 300 ml/h	4.6 ± 1.4	3000 rpm no oxygenator 300 ml/h	1.6 ± 1.1	*p* < 0.0001
4000 rpm oxygenator 300 ml/h	6.1 ± 1.5	4000 rpm no oxygenator 300 ml/h	2.9 ± 0.7	*p* < 0.0001

## Discussion

Echocardiography is an important investigation in patients supported with ECMO. Transthoracic echocardiography is typically the first line cardiac imaging investigation in these patients [13]. However, up to 25 % of transthoracic echocardiograms may be non-diagnostic due to adverse ultrasound scanning conditions. These include patients being immobile, supine and attached to a ventilator, issues with accessing suitable acoustic windows and sub-optimal lighting conditions [14, 15]. Contrast-specific echocardiographic imaging modalities can help overcome these limitations and increase the diagnostic yield of echocardiography. However, echocardiographic contrast agents are hydrodynamically labile microspheres and likely to be prone to destruction within an ECMO circuit. There are multiple factors which can influence contrast microsphere stability, including the insonicating ultrasound power (mechanical index), ambient temperature, sheer forces and also the particle pressure of gases [16]. These contrast microspheres are likely to be prone to destruction within an ECMO circuit. In our research, this was demonstrated by a decrease in microbubble concentration at both infusion rates, with and without an oxygenator in situ and at all pump head speeds. Despite the statistically significant decrease in signal intensity through the circuit without an oxygenator, the smaller magnitude of drop suggests that the oxygenator contributes to a significantly greater degree of bubble destruction than in the pump head alone (75 versus 25 %, respectively). To our knowledge, this is the first study to evaluate the site and quantify the degree of contrast microbubble destruction within an ECMO circuit.

An ECMO circuit in clinical use consists of access and return cannulae, biocompatible tubing to transport the blood between the patient and ECMO pump, a controller to adjust flows, a blood warmer and an oxygenator for gas exchange [1]. There are three likely sites of contrast microbubble destruction within an ECMO circuit: the access and return points, the pump head and finally, the oxygenator. The access and return points usually consist of large bore cannulae, of which there are numerous designs [17]. The flow within the main body of these cannulae is laminar. At the points of blood entering the ECMO circuit at the tip of the access cannula and blood exiting the circuit in the return cannula, the flow would transform from laminar to turbulent. This turbulent flow would have an impact on microsphere destruction. Flow is also turbulent at the edge of the blood pool and adjacent to the inner surface of the cannulae or tubing. This is termed boundary layer turbulence [17]. Along the whole length of the ECMO circuit tubing (not only just the cannulae), this boundary layer turbulence will exist and also contribute to the destruction of the contrast microbubbles. However, the degree of turbulence here is likely to be significantly less than in the pump head.

Contrast microbubble destruction is more likely to occur at the pump head and the oxygenator. At these sites, there is very high velocity flow, significant pressure differentials and non-laminar, turbulent flow, all of which increase the shear stress on microbubbles. The pump head used in this ECMO circuit was a Rotaflow centrifugal pump. This has a mechanical, low-friction bearing point and is magnetically rotated. The priming volume is 32 ml, the surface area is only 0.19 cm^2^ and the rotor diameter is 50 mm and is constructed with polycarbonate [18]. The internal rotor spins at a rate which can be adjusted on the external controller, with spin rates in the clinical environment often in the range of 2500–5000 rpm. This spinning generates a constrained vortex and hence a pressure differential across the pump head [19].

Contrast microsphere destruction may occur within the pump head due to several factors. Direct contact with a rapidly rotating mechanical structure within the pump head is likely to lead to microbubble destruction. Additionally, the non-physiologic and rapid centrifugal flow within the pump head is likely to directly contribute to microbubble destruction. The high pressure drop across the pump head would contribute to increased destructive shear forces. In this research, there was a statistically significant decrease in microbubble concentration across the pump head, with the most likely mechanism being destruction from shear forces and the turbulent flow. With increasing pump head speeds, there was an increase in microsphere destruction (except for the increment from 1000 to 2000 rpm). With higher spin rates, the destructive forces within the rotor housing would be higher (especially due to increased flow turbulence and greater pressure differentials) and this would result in a greater degree of microsphere destruction within the pump head. Whist one could anticipate a linear correlation between microsphere destruction and all pump head speeds, it could be postulated that the absolute destructive forces at these lower pump head speeds (1000 to 2000 rpm) may not be sufficiently different to be translated into a significant difference in microsphere destruction at this lower revolutions per minute increment. Unexpectedly, however, the degree of microbubble destruction caused by the pump head was significantly less than that caused by the oxygenator.

**Fig. 6:**
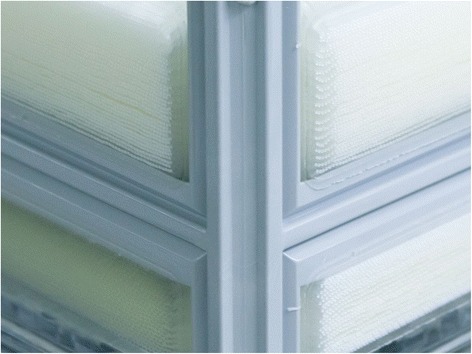
Quadrox oxygenator during construction. Note the multiple layers of hollow fibre mats interposed repeatedly. Image reproduced with permission (Maquet Cardiopulmonary AG, Germany)

**Fig. 7:**
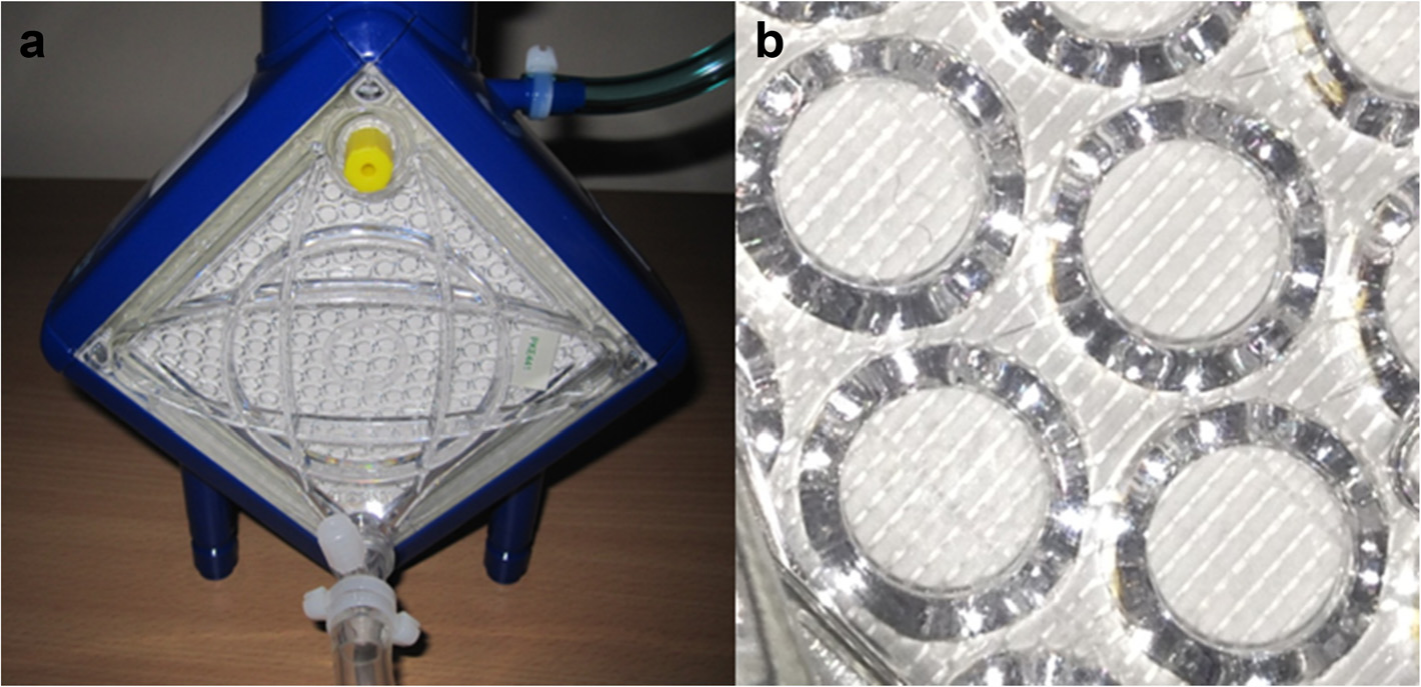
**a** The Quadrox PLS oxygenator and **b** the polymethylpentene fibre mats within the oxygenator

Oxygenators are used within standard cardiopulmonary bypass machines and within ECMO circuits. They are one of the key components within an extracorporeal support circuit. Oxygenators have the dual purpose of enabling gas exchange and providing thermoregulation of the blood [20, 21]. Contemporary ECMO oxygenators can be classified as silicone membrane oxygenators, microporous hollow fibre oxygenators and true membrane hollow fibre oxygenators [22]. The commonest type currently used in ECMO, including the Quadrox PLS, is a true membrane hollow fibre oxygenator. These oxygenators consist of mats of hollow fibres constructed of polymethylpentene. The fibres are laid out in parallel mats which are then interposed repeatedly. This is demonstrated in Fig. 6. The priming volume of the Quadrox PLS oxygenator is 250 ml, with blood flow tolerance of between 0.5 and 7.0 l/min. The effective surface area for gas exchange is 1.8 m^2^ [18]. Figure 7a shows the Quadrox PLS oxygenator, and Fig. 7b demonstrates the polymethylpentene fibre mats within the oxygenator.

In this research, there was a significantly greater level of contrast microsphere destruction in a circuit with an oxygenator. This implies that the oxygenator played an important role in contrast destruction within an ECMO circuit. Despite the presence of significant microbubble destruction during passage through an ECMO circuit, there was still a strong backscatter signal from residual contrast, even with an oxygenator in situ. This suggests that contrast-enhanced transthoracic echocardiography would most likely be technically feasible in this patient population, with adjustment of the infusion rate to achieve an optimal contrast-enhanced image. As there was no significant difference in mean signal reduction across the circuits between the two infusion rates, this suggests that infusion rate does not have a significant impact on the degree of bubble destruction within the ECMO circuit, though it may impact baseline image quality. Consequently, the clinician can adjust the baseline infusion rate with the knowledge that higher infusion rates would not cause relatively greater degrees of bubble destruction, which may then adversely affect image quality. Contrast-enhanced echocardiography has been shown to be possible in an ovine model of ECMO and, in the clinical setting, with an oxygenator in the circuit [23–25].

One important parameter affecting shear stress is the pressure drop across a structure. At peak flows, the pressure drop across an ECMO pump head can be as high as 900 mmHg. However, at maximal flows, the pressure drop across an oxygenator is much lower, approximately 75 mmHg. This would imply that the shear forces generated within the pump head would contribute to a greater degree of bubble destruction than within the oxygenator.

However, the results of this study showed that there was a significant difference (increase) in microbubble destruction between a full circuit (with an oxygenator) versus a circuit with no oxygenator, and this occurred at all pump head speeds. This suggested that the presence of an oxygenator was a more important predictor of microbubble destruction than the actual pump head speed. There are several mechanisms that may explain this unexpected increase in microbubble destruction with the oxygenator in situ. Firstly, between the entry and exit points of an oxygenator, the flow path may be complex and involve numerous acute changes in direction, with repeated contact against the rigid polymethylpentene fibres. This could increase the shear stress on microbubbles and hence promote destruction. As the oxygenator is designed to maximise mass transfer within a relatively small and confined space, the repeatedly interposed fibre mats would act as recurrent sites for microbubble trauma.

Alternative mechanisms for oxygenator-induced microsphere destruction may not directly involve shear forces but be related to the function of gas trapping and hydrophobic interactions. Other than gas transfer, oxygenators also provide a gas/air trapping protective function. The inlet and outlet points of an oxygenator are specifically at its base to enable any air within the circuit, which would ‘rise’, to be removed via the outlet at the top of the oxygenator and hence eradicated from the circuit. Whilst the comparative buoyancy properties of Definity® microspheres in normal saline versus blood are not known, these microspheres are buoyant and will rise to the surface in a liquid [26]. As such, it is conceivable that some of the delivered contrast agent is gas trapped within the oxygenator, in a similar way that air bubbles would be trapped.

Hydrophobic interactions between the contrast microsphere and hollow fibre membranes within an oxygenator (and other components within an ECMO circuit) may also contribute to microsphere destruction. These hydrophobic interactions have been shown to be of importance during ECMO pharmacokinetic studies, where up to 90 % of some drugs may be removed by the circuit [27, 28]. Common reasons for drug pharmacokinetics being influenced by ECMO in vivo include circuit sequestration, increased volume of distribution, and decreased drug elimination [29]. Whilst not all of these are relevant to our ex vivo circuit, contrast microspheres are hydrophobic but not lipophilic. As such, direct interactions between these hydrophobic microspheres and the hydrophobic polymethylpentene hollow fibres may also result in trapping of the microspheres within the oxygenator.

### Study limitations

This study was performed in an ex vivo ECMO circuit where the differential destruction of the pump head and oxygenator were assessed in controlled laboratory conditions. Whilst the circuit equipment is the same as used clinically, there are numerous important differences. The main difference was that there was no patient component that would contribute to microbubble destruction. Also, in light of this, there were no access or return cannulae located within the ex vivo circuit. Flow through these cannulae also contributes to microbubble destruction. There are numerous sites for contrast microbubble destruction in the ECMO circuit, including the conduit tubing. This research focussed on the oxygenator and pump head components. It was not possible to isolate the relative contribution of boundary turbulent flow related to this tubing, which is an important component of an ECMO circuit. Also, whilst unlikely to be clinically relevant to microbubble destruction, this ex vivo circuit was primed with normal saline, which has a viscosity approximately one third that of blood. Ideally, blood would have been the preferable product to prime the ECMO circuit. However, the constraints of our research resulted in access to saline for the circuit priming. However, the results may be still meaningful as there were significant identifiable differences in the contrast concentrations between the two circuits, and as saline was used in both circuits, it would not have accounted for this difference in signal intensity.

The signal intensity measured within the 5-mm^2^ ROI was a mean of 78 values over 2 s (as these contrast-specific images were acquired at a frame rate of 39 Hz) which varied around this mean. The signal intensity was not constant over these 2 s clips within this ROI despite a constant infusion. This slight signal intensity variation would be most likely due to the rapid flow of contrast microbubbles mixed through the larger volume of 0.9 % sodium chloride priming solution traversing the ECMO circuit tubing. This is demonstrated in Additional file 1: Video 1 and Additional file 2: Video 2. Whilst contrast microspheres have a short half-life, the circuit was not flushed and re-primed after removal of the oxygenator. As such, there may have been a small quantity of residual contrast within the circuit for the commencement of the second circuit. However, it was felt this would not have a significant effect due to the high concentration following reinfusion and the assessment of relative contrast destruction across the circuit. Finally, this research analysed one type of ECMO circuitry (Quadrox PLS Oxygenator and Rotaflow pump head). Other ECMO circuitry components are available, and our results would not be interchangeable with other circuits.

## Conclusions

There was a significant decrease in contrast microsphere concentration during the passage through an ex vivo ECMO circuit. This phenomenon occurred regardless of pump head speed, but higher pump head speeds resulted in greater microsphere destruction. The presence of an oxygenator within the ECMO circuit contributed to microsphere destruction at a significantly greater level than the pump head alone. Translation to the bedside suggests that clinicians should be cognisant of the relationship between ECMO circuit configurations, pump head speed and contrast destruction when performing contrast-enhanced transthoracic echocardiography.

## Additional files

Additional file 1: Video 1. Two second movie clip of the ECMO circuit pre-pump at 4000 RPM. (AVI 2000 kb) Additional file 2: Video 2. Two second movie clip of the ECMO circuit post-pump at 4000 RPM. (AVI 2000 kb)
